# Reduced risk of prostate cancer in childless men as compared to fathers: a systematic review and meta-analysis

**DOI:** 10.1038/srep19210

**Published:** 2016-01-11

**Authors:** Yeqing Mao, Xin Xu, Xiangyi Zheng, Liping Xie

**Affiliations:** 1 Department of Urology, First Affiliated Hospital, School of Medicine, Zhejiang University, Hangzhou, 310003, China

## Abstract

The previously reported association between fatherhood status and prostate cancer risk was controversial. We carried out the present meta-analysis of all relevant studies to summarize evidence on this association. A comprehensive literature search of studies was performed in PubMed, Web of Science, and the Chinese National Knowledge Infrastructure (CNKI) databases, covering all the papers published from their inception to September 2015. The combined risk estimates with 95% confidence intervals (CIs) were calculated using a random effects model. Heterogeneity and publication bias were also evaluated. A total of 11 studies were finally included in this meta-analysis. We found a significantly reduced risk of prostate cancer associated with being childless (OR 0.91, 95% CI 0.87–0.96). There was statistically significant heterogeneity across the studies (*P* < 0.001, *I*^*2*^ = 88.2%). In summary, this meta-analysis supports that being fatherless is associated with a lower risk of prostate cancer. Because of the substantial heterogeneity and residual confounding, using other study designs to further explore this association and the underling mechanism is warranted.

Prostate cancer is the second most common form of cancer in males worldwide, with an expected 1,111,700 new cases and 307,500 deaths in 2012^1^. Age, race/ethnicity, and family history of prostate cancer are the most clearly established risk factors for prostate cancer^2^. A greater prevalence of prostate cancer in western countries^3^ and migrant data implicate lifestyle and socio-environmental risk factors^4^. Emerging evidence indicates specific vegetables^5^,^6^, physical activity^7^, and obesity^8^ may be related with the incidence of prostate cancer, although controversies still exist.

Recently, several researchers have explored whether fatherhood status is a potential risk factor for prostate cancer with conflicting results. A Swedish case–control study^9^, a prospective US cohort study^10^, and a Danish cohort study^11^ suggested that childless men had a lower risk of prostate cancer compared with fathers. They have hypothesized that androgen status may account for this relationship, as infertile men may have impaired testicular function^12^ and prostate cancer is known to be testosterone dependent^13^. In contrast, several other studies^14^,^15^,^16^ failed to confirm the observed inverse association between fatherhood and prostate cancer risk. Rosenblatt *et al.*^17^ even reported a significantly positive association. Given the controversial findings as discussed above, we performed the present meta-analysis to summarize evidence on the association between fatherhood status and the risk of prostate cancer.

## Results

### Literature search and study characteristics

**Figure 1:**
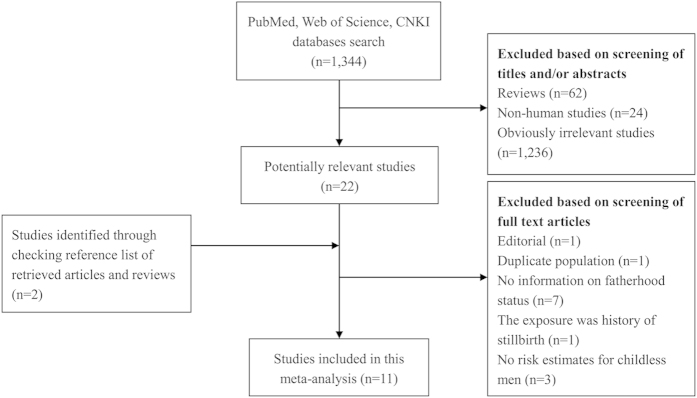
Process of literature search and study selection.

The detailed processes of literature search are presented in Fig. 1. Eleven studies^9^,^10^,^11^,^14^,^15^,^16^,^17^,^18^,^19^,^20^,^21^ (five case-control studies, five cohort/nested case-control studies, and one pooled analysis) were finally included in this meta-analysis of the association between fatherhood status and prostate cancer risk. All of these studies were published between 2001 and 2013. Types of exposure included being childless (n = 8) and infertility (n = 3). Except the pooled analysis study, the characteristics of ten individual studies were summarized as follows: study regions included North America (n = 4), Europe (n = 5), and New Zealand (n = 1); the number of cases ranged from 168 to 117,328, with a total of 182,012; study quality scores ranged from 5 to 8, with a mean of 7.1; the number of studies adjusting for age, marital status, and education were ten, five, and four, respectively. Table 1 presents the main characteristics of each study included in this meta-analysis.

### Overall and subgroup analyses

**Figure 2:**
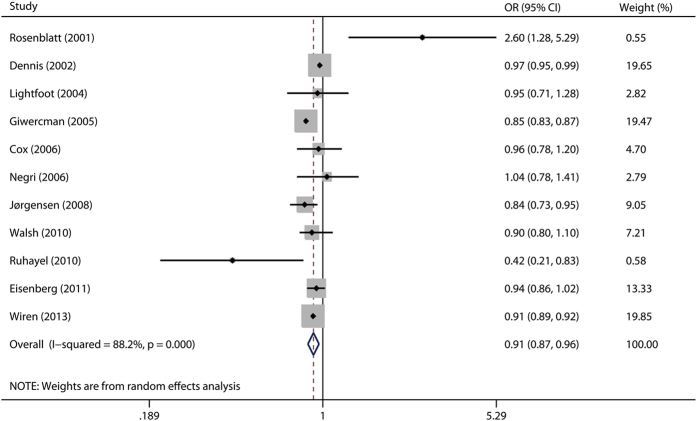
A forest plot showing risk estimates from case–control and cohort studies estimating the association between fatherhood status and prostate cancer risk.

The multivariable-adjusted ORs of childless men versus fathers, for each study and for the combination of all the studies, are presented in Fig. 2. We found a significantly decreased risk of prostate cancer associated with being childless (OR 0.91, 95% CI 0.87–0.96). There was statistically significant heterogeneity across the studies (*P* < 0.001, *I*^*2*^ = 88.2%).

Then we conducted stratified analyses by study design, geographical region, number of cases, type of exposure, marital status, and education (Table 2). In the subgroup analysis by study design, the observed association was more pronounced in the cohort/nested case-control studies (OR 0.90, 95% CI 0.85–0.95) than in the case–control studies (OR 0.99, 95% CI 0.82–1.19). When we stratified by geographical region, the ORs (95% CI) were 0.98 (0.82–1.16), 0.87 (0.82–0.93), and 0.96 (0.78–1.20) for North America, Europe, and Oceania, respectively. When separately analyzed by number of cases, more significant association was observed in large studies (OR 0.89, 95% CI 0.84–0.93) compared with that in small studies (OR 0.96, 95% CI 0.78–1.19). In the subgroup analyses separated by type of exposure, the ORs (95% CI) were 0.98 (0.45–2.12) for male infertility and 0.91 (0.87–0.96) for childless men. When stratifying by marital status, the link was more pronounced in the studies adjusted for marital status (OR 0.90, 95% CI 0.85–0.97) than in the studies not adjusted for marital status (OR 0.93, 95% CI 0.81–1.08). In the subgroup analysis by education, the ORs (95% CI) were 0.92 (0.85–1.00) for studies adjusted for education and 0.90 (0.81–0.99) for studies not adjusted for education.

### Evaluation of heterogeneity

**Figure 3:**
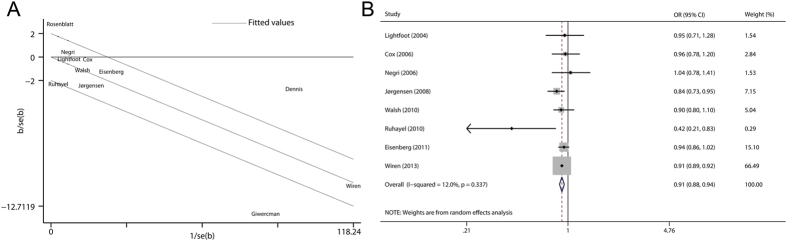
Evaluation of heterogeneity. (**A**) Galbraith plot analysis was used to detect potential sources of heterogeneity; (**B**) Pooled risk estimate with its 95% CI for the association between fatherhood status and prostate cancer risk after removing studies that contribute most to heterogeneity.

In this study, the *Q* test and the *I*^*2*^ index were adopted to examine the heterogeneity among included studies. As shown in Fig. 2, statistically significant heterogeneity was observed across the studies (*P* < 0.001 for heterogeneity, *I*^*2*^ = 88.2%). Then the Galbraith plot was used to detect the potential sources of heterogeneity. As shown in Fig. 3A, the studies performed by Rosenblatt *et al.*^17^, Dennis *et al.*^18^, and Giwercman *et al.*^9^ might contribute to the heterogeneity. The omission of these publications markedly reduced the heterogeneity (*P* = 0.337 for heterogeneity, *I*^*2*^ = 12.0%) while the overall association remained significant (OR 0.91, 95% CI 0.88–0.94) (Fig. 3B).

### Sensitivity analysis and cumulative meta-analysis

**Figure 4:**
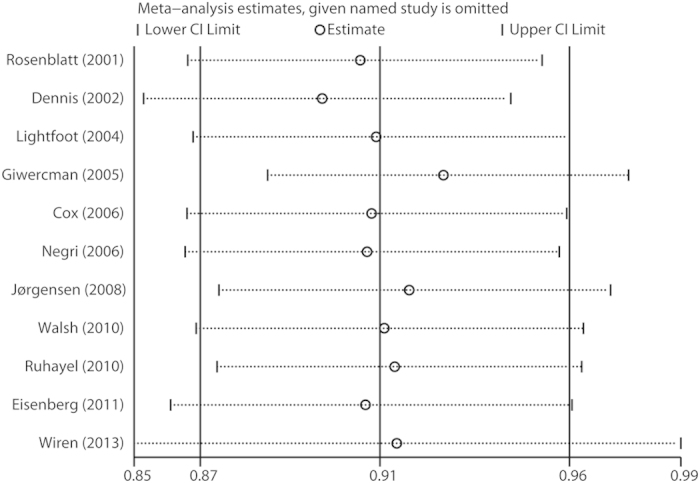
Sensitivity analysis was performed by excluding each study in turn and recalculating the pooled estimates.

In the sensitivity analysis, the influence of each study on the summary effect estimate was evaluated by repeating the meta-analysis after removing one study at a time. As shown in Fig. 4, the pooled ORs were not considerably affected by omitting any individual study, which indicated that our results were robust. Cumulative meta-analysis was performed by sorting the included studies based on publication date. Supplementary Figure S1 shows the results from the cumulative meta-analysis of the association between fatherhood status and prostate cancer risk in chronologic order.

### Publication bias

**Figure 5:**
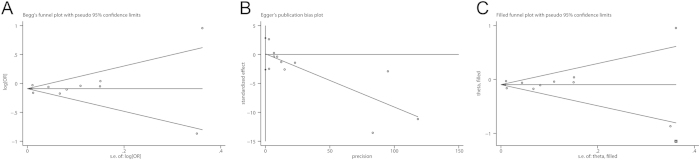
Evaluation of publication bias. (**A**) Begg’s test (rank correlation method); (**B**) Egger’s test (linear regression method); (**C**) trim-and-fill analysis.

There was no evidence of significant publication bias with Begg’s test (Fig. 5A, *P* = 0.533). However, Egger’s test suggested the existence of publication bias (Fig. 5B, *P* = 0.003). The trim-and-fill method identified one possible (imputed) missing study (Fig. 5C), which did not alter the findings substantially (OR 0.91, 95% CI 0.86–0.96).

## Discussion

This study summarizes and quantifies the current evidence on the association between fatherhood status and prostate cancer risk in a meta-analysis of observational studies, including five case–control studies, five cohort/nested case-control studies, and one pooled analysis study. To the best of our knowledge, this is the first meta-analysis evaluating the association between fatherhood status and prostate cancer risk. The results indicated that being fatherless was inversely associated with the incidence of prostate cancer (OR 0.91, 95% CI 0.87–0.96).

Androgen status may mediate the link between fatherhood status and prostate cancer risk. Infertile men have lower levels of testosterone and serum testosterone to estradiol ratios compared with fertile men^22^. Low circulating levels of testosterone have been suggested to be associated with a reduced risk of prostate cancer^23^, although the evidence from observational studies remains controversial^24^. In addition, inhibition of dihydrotestosterone (DHT) serum levels by use of 5α-reductase inhibitors markedly reduced the risk of prostate cancer in two large randomized clinical trials^25^,^26^.

It is worth noting that fatherhood status as a proxy for male fertility are hampered by the fact that the fatherhood status is influenced by various factors comprising fertility of the man, fertility of the partner, opportunity to start a family, and desire to have children. In subgroup analysis by marital status, the association was still statistically significant in the studies adjusted for marital status (OR 0.90, 95% CI 0.85–0.97). Thus, the presence of confounding from unmarried men could be partially ruled out. However, we were not able to assess the differential misclassification from men’s reproductive intent and fertility of their partners.

Another important confounding factor was the difference in healthcare-seeking patterns, as married and better educated men may have a higher uptake of PSA testing^27^. In this study, we conducted stratified analysis by education and the pooled estimate remained consistently significant in studies adjusted for education. Nevertheless, as the majority of the included studies did not provide the information of PSA testing, we could not evaluate the role of PSA testing in the association between fatherhood status and incidence of prostate cancer. Therefore, there are reasons to believe that the beneficial effects of being fatherless were at least in part due to unmeasured and residual confounding.

Our study had some strengths. A total of 182,012 prostate cancer cases were included in this meta-analysis, which enhanced the statistical power and provided more reliable estimates. The estimates from fully adjusted models in each study were used in this study to minimize potential confounding. Various subgroup analyses and sensitivity analyses were carried out to assess the robustness of the results.

Our study also had several important methodological limitations. First, substantial heterogeneity was observed across individual studies (*P* < 0.001 for heterogeneity, *I*^*2*^ = 88.2%), which might distort the pooled estimates. Through the Galbraith plot, we detected the studies that potentially contributed to the heterogeneity. After removing these studies, the combined estimate remained significant (OR 0.91, 95% CI 0.88–0.94) without obvious heterogeneity (*P* = 0.337 for heterogeneity, *I*^*2*^ = 12.0%), which indicated that the heterogeneity didn’t have a material impact on our conclusion. Second, publication bias was detected by Egger’s test (*P* = 0.003), although the trim-and-fill analysis did not alter the findings substantially. Small negative studies were less likely to be published and we were not able to include some gray literature, such as conference abstracts and studies reported in languages other than English or Chinese. Third, half of the included studies adopted case-control design, which may introduce the possibility of select bias and recall bias. However, the observed association was robust and consistent in the subgroup of cohort/nested case-control studies (OR 0.90, 95% CI 0.85–0.95). Fourth, there were vast differences in size of included studies, ranging from 168 to 117,328. The pooled estimate was vulnerable to the results of large studies, such as Giwercman *et al.*’s and Wiren *et al.*’s studies^9^,^21^. In addition, these two largest studies^9^,^21^ were both nationwide in Sweden and had an overlap of cases (between 1991 and 1998), which may lead to biased estimates.

Overall, this meta-analysis supports that being fatherless is associated with a low incidence of prostate cancer. Because of the substantial heterogeneity and residual confounding, using other study designs to further explore this association and the underling mechanism is warranted.

## Methods

### Literature search

We performed a comprehensive literature search of studies in PubMed, Web of Science, and the Chinese National Knowledge Infrastructure (CNKI) databases, covering all the papers published from their inception to September 2015. We adopted the following search algorithm: (fertility OR infertility OR infertile OR fatherhood OR father OR childless OR child OR children OR offspring OR marriage OR partner OR sex OR sexual OR sexually OR intercourse OR coitus) AND (prostate OR prostatic) AND (cancer OR neoplasm OR tumor OR malignancy) AND (cohort OR case-control OR case control). No language limitation was applied. We evaluated all retrieved publications carefully by examining their titles and abstracts, and the full texts of studies potentially matching the eligible criteria were further checked. We also reviewed reference lists of articles and reviews to identify any additional relevant studies. The present systematic review was planned, conducted, and reported in adherence to standards of quality for reporting meta-analyses^28^.

### Study selection

The studies included in this meta-analysis met all of the following criteria: (*i*) the exposure of interest was being childless or infertility. Infertility is defined as the absence of a live birth for men who desire a child. Considering infertile men generally have no children, the studies with exposure of male infertility were also included in the pooled analysis; (*ii*) the outcome of interest was prostate cancer; (*iii*) study design was case-control or cohort; and (*iv*) the effect size estimates with their corresponding 95% confidence intervals (CIs) were provided (or data were sufficient to calculate these values). Whenever multiple articles from the same study population were available, the study with the largest number of cases and most applicable information was included in this meta-analysis. In particular, we included a pooled analysis of 18 studies, rather than separate articles, since part of these articles are non-English literature or lack sufficient data to calculate the effect size estimate.

### Quality assessment

The quality of each study was evaluated by two authors (Y.M. and X.X.) using the Newcastle-Ottawa Scale (NOS) (http://www.ohri.ca/programs/clinical_epidemiology/oxford.asp). This scale has three categories: Selection (four items), Comparability (one item), and Exposure for case-control studies or Outcome for cohort studies (three items). A study can be awarded a maximum of one star for each numbered item within the Selection and Exposure/Outcome categories. A maximum of two stars can be given for Comparability. The stars are then added up to obtain the total quality score.

### Data extraction

The following information were extracted from each study: first author’s name, year of publication, study design, the country in which the study was carried out, sample size, age of study population, exposure assessment, duration of follow-up for cohort/nested case-control studies, adjusted risk estimates with their 95% CIs, and matched or adjusted factors in the design or data analysis. Data extraction was performed independently by two authors (Y.M. and X.X.) using a predesigned data collection form. Discrepancies were resolved by group consensus and consulting a third reviewer.

### Statistical methods

Considering that prostate cancer is a rare disease, the relative risk (RR) was assumed to be nearly equal to the odds ratio (OR), and the OR was used as the study outcome. The combined OR with its 95% CI was calculated to assess the strength of the association between fatherhood status and prostate cancer risk^29^. For studies which reported separate risk estimates for different number of children (e.g., one child, two children, three children, and so on), we pooled these risk estimates within each study, weighted by inverse of the variance^30^. Stratified analyses were carried out by study design, geographical region, number of cases, type of exposure, marital status, and education.

The heterogeneity among included studies was tested by the *Q* statistic and the *I*^*2*^ score^31^. Statistical significance of heterogeneity was set at 0.1. The value of *I*^*2*^ was used to describe the degree of heterogeneity (*I*^*2*^ < 25% no heterogeneity; *I*^*2*^ = 25–50% moderate heterogeneity; *I*^*2*^ > 50% large or extreme heterogeneity). Galbraith plot^32^ was used to detect the possible sources of heterogeneity. A re-analysis was carried out after removing the studies possibly leading to the heterogeneity.

Sensitivity analysis was conducted by repeating the meta-analysis after the omission of every study in turn to assess the effect of each study on the combined estimate. Cumulative meta-analysis was also performed by sorting the studies based on publication year.

Potential small-study bias was evaluated by Begg’s test (rank correlation method)^33^, Egger’s test (linear regression method)^34^, and trim-and-fill method^35^. If *P* < 0.05, results were considered statistically significant. All of the statistical analyses were conducted with STATA 11.0 (StataCorp, College Station, TX), using two-sided *P* values.

## Additional Information

**How to cite this article**: Mao, Y. *et al.* Reduced risk of prostate cancer in childless men as compared to fathers: a systematic review and meta-analysis. *Sci. Rep.* **6**, 19210; doi: 10.1038/srep19210 (2016).

## Supplementary Material

Supplementary Information

## Figures and Tables

**Table 1:** Characteristics of the studies included in meta-analysis of association between fatherhood status and prostate cancer risk.

Study	Population information	Exposure assessment	Outcome assessment	Study design, cases/cohort (controls)	Age, yr	Follow-up, yr	Type of exposure (reference group)	OR/RR (95% CI)	Matched or adjusted factors	NOS score
Rosenblatt *et al.*, 2001, US	A population-based study performed in King County, Washington between 1993 and 1996	Interview	Cancer registry	Case-control, 753/703	40–64	NA	Infertile men (fertile men)	2.60 (1.28–5.29)	Age, race, family history of prostate cancer, and number of PSA tests in the past 5 years	7
Dennis *et al.*, 2002, Multi-country	A pooled analysis of 18 studies based on a linear dose-response model	NA	NA	NA	NA	NA	Childless men (fathers)	0.97 (0.95–0.99)	NA	NA
Lightfoot *et al.*, 2004, Canada	A population-based study conducted from 1995 to 1999 in northeastern Ontario	Mailed questionnaire	Cancer registry	Case-control, 760/1,632	45–84	NA	Childless men (fathers)	0.95 (0.71–1.28)	Age	5
Giwercman *et al.*, 2005, Sweden	A population-based study with retrospective ascertainment of cases occurring in Sweden between 1958–98	Multi-Generation Register	Cancer registry	Case-control, 48,850/48,850	NA	NA	Childless men (fathers)	0.85 (0.83–0.87)	Age at diagnosis, year and county of birth	7
Cox *et al.*, 2006, New Zealand	Histology reports of men diagnosed with prostate cancer between 1 April 1996 and 31 December 1998	Interview	Cancer registry	Case-control, 664/892	66.3 (40–74)	NA	Childless men (fathers)	0.96 (0.78–1.20)	Age	7
Negri *et al.*, 2006, Italy	A case-control study conducted between 1991 and 2002 in 4 Italian areas	Interview	Histologically confirmed	Case-control, 1,294/1,451	66 (46–74)	NA	Childless men (fathers)	1.04 (0.78–1.41)	Age, center, marital status, age at marriage, education, BMI, physical activity, smoking, alcohol intake, and family history of prostate cancer	8
Jørgensen *et al.*, 2008, Denmark	A cohort comprising all Danish men born between 1935 and 1988	Civil Registration System	Cancer registry	Cohort, 3400/NA	60 (26–68)	1968–2003	Childless men (fathers)	0.84 (0.73–0.95)	Age, period, and marital status	7
Walsh *et al.*, 2010, US	A cohort of couples who sought evaluation for infertility in California	Evaluation for infertility	Cancer registry	Cohort, 168/22,562	>18	11.4	Infertile men (general population)	0.90 (0.80–1.10)	Age	7
Ruhayel *et al.*, 2010, Sweden	Malmö Diet and Cancer Study	Self-administered questionnaire	Cancer registry	Nested case-control, 661/661	74.3 ± 5.7	1991–2006	Infertile men (fertile men)	0.42 (0.21–0.83)	Age, previous history of urogenital infections, height, weight, waist circumference, education, marital status, smoking status, and country of birth	8
Eisenberg *et al.*, 2011, US	NIH-AARP Diet and Health Study	Self-report	Cancer registry	Cohort, 8,134/161,823	63 (50–71)	1995–2003	Childless men (fathers)	0.94 (0.86–1.02)	Age, education, race, marital status, DRE screening, BMI, smoking status, and family history of prostate cancer.	7
Wiren *et al.*, 2013, Sweden	A case–control study in Prostate Cancer data Base Sweden 2.0, a nationwide, population-based cohort	Multi-Generation Register	Cancer registry	Nested case-control, 117,328/562,644	NA	1991–2009	Childless men (fathers)	0.91 (0.89–0.92)	Birth year, county of residence, socioeconomic status, education, and marital status	8

PSA, prostate-specific antigen; NOS, Newcastle-Ottawa Scale; yr, year; BMI, body mass index; DRE, digital rectal examination; NIH-AARP, National Institutes of Health-American Association of Retired Persons; NA, not available.

**Table 2:** Subgroup analyses of the association between fatherhood status and prostate cancer risk.

Subgroup	Included studies	Pooled RR (95% CI)	*P*	Heterogeneity		
				*Q*	*I*^*2*^ (%)	*P*
Total	11^9^,^10^,^11^,^14^,^15^,^16^,^17^,^18^,^19^,^20^,^21^	0.91 (0.87–0.96)	0.001	85.04	88.2	<0.001
Study design						
Cohort/nested case-control	5^10^,^11^,^19^,^20^,^21^	0.90 (0.85–0.95)	<0.001	6.85	41.6	0.144
Case-control	5^9^,^14^,^15^,^16^,^17^	0.99 (0.82–1.19)	0.882	12.95	69.1	0.012
Geographical region						
North America	4^10^,^14^,^17^,^20^	0.98 (0.82–1.16)	0.807	8.19	63.4	0.042
Europe	5^9^,^11^,^16^,^19^,^21^	0.87 (0.82–0.93)	<0.001	27.94	85.7	<0.001
Oceania	1^15^	0.96 (0.78–1.20)	0.710	NA	NA	NA
No. of cases						
≥3000	4^9^,^10^,^11^,^21^	0.89 (0.84–0.93)	<0.001	23.89	87.4	<0.001
<3000	6^14^,^15^,^16^,^17^,^19^,^20^	0.96 (0.78–1.19)	0.737	13.95	64.2	0.016
Type of exposure						
Male infertility	3^17^,^19^,^20^	0.98 (0.45–2.12)	0.961	13.29	85.0	0.001
Childless men	8^9^,^10^,^11^,^14^,^15^,^16^,^18^,^21^	0.91 (0.87–0.96)	0.001	71.74	90.2	<0.001
Adjusted for marital status						
Yes	5^10^,^11^,^16^,^19^,^21^	0.90 (0.85–0.97)	0.004	7.62	47.5	0.107
No	5^9^,^14^,^15^,^17^,^20^	0.93 (0.81–1.08)	0.342	11.66	65.7	0.020
Adjusted for education						
Yes	4^10^,^16^,^19^,^21^	0.92 (0.85–1.00)	0.038	6.18	51.5	0.103
No	6^9^,^11^,^14^,^15^,^17^,^20^	0.90 (0.81–0.99)	0.031	11.72	57.3	0.039

No., number; RR, relative risk; CI, confidence interval; NOS, Newcastle-Ottawa Scale; NA, not available.
