# Supplementary material for: Reduced risk of prostate cancer in childless men as compared to fathers: a systematic review and meta-analysis
Source: Sci Rep. 2016 Jan 11;6:19210. doi: 10.1038/srep19210 (PMC4707492; doi:10.1038/srep19210)

Reduced risk of prostate cancer in childless men as compared to fathers: a systematic review and meta-analysis

Yeqing Mao, Xin Xu, Xiangyi Zheng, and Liping Xie

Supplementary Figure S1. Cumulative meta-analysis was performed based on publication year.

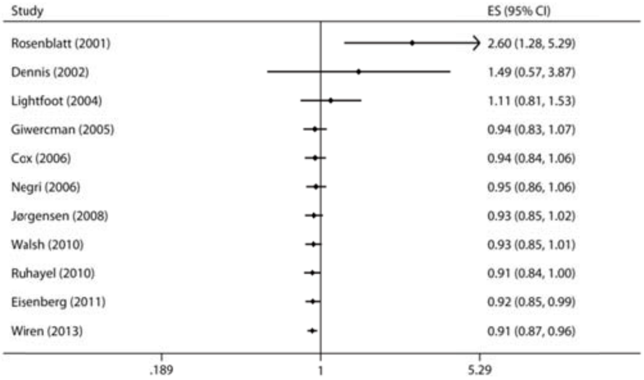

Supplement: Supplementary Information [file srep19210-s1.pdf]
